# The complete mitochondrial genome sequences of Japanese earthworms *Metaphire hilgendorfi* and *Amynthas yunoshimensis* (Clitellata: Megascolecidae)

**DOI:** 10.1080/23802359.2020.1830728

**Published:** 2021-03-18

**Authors:** Akira Seto, Hayato Endo, Yukio Minamiya, Masaru Matsuda

**Affiliations:** a Graduate School of Regional Development and Creativity, Utsunomiya University, Utsunomiya, Japan; b Center for Bioscience Research and Education, Utsunomiya University, Utsunomiya, Japan; c Oyama High School, Oyama, Japan; d Tochigi Prefectural Museum, Utsunomiya, Japan

**Keywords:** Earthworms, *Metaphire hilgendorfi*, *Amynthas yunoshimensis*, complete mitochondrial genome

## Abstract

Many studies have reported the complete mitochondrial genome sequences of Chinese Megascolecidae earthworms, however, there have been no reports on sequences originating from Japanese Megascolecidae earthworms. In this study, we determined complete mitochondrial genome sequences of two Japanese earthworms belonging to the *Pheretima* complex within the Megascolecidae family. *Metaphire hilgendorfi* is one of the most common earthworms in Japan and *Amynthas yunoshimensis* was found to be morphologically similar to *M. hilgendorfi*. The complete mitochondrial genomes of *M. hilgendorfi* (15,186 bp; LC573968) and *A. yunoshimensis* (15,109 bp; LC573969) contained typical 13 protein coding genes, 22 tRNA genes, and 2 rRNA genes. The phylogenetic analysis revealed that these two species were sister species. Therefore, our findings will further contribute to phylogenetic and genetic diversity analyses of Megascolecidae earthworms.

Analysis of the complete mitochondrial genome of Megascolecidae family earthworms has been given special focus. After the complete mitochondrial genome sequence of *Lumbricus terrestris* was determined (Boore and Brown 1995), the sequences of 28 earthworm species belonging to 4 families have now been published (Zhang et al. 2014a, 2014b, 2015, 2016, 2019; Wang et al. 2015; Conrado et al. 2017; Hong et al. 2017; Shekhovtsov and Peltek 2019). Most of them are Chinese Megascolecidae earthworms, of which 22 species have been reported of belonging to the *Pheretima* complex. However, the complete mitochondrial genome sequences of Japanese earthworms have not been reported until date.

The *Pheretima* complex is one of the largest earthworm groups within the Clitellata, with 14 genera including approximately 1100 validated species (DriloBase, http://taxo.drilobase.org/index.php?title=List_of_taxa/Megascolecidae, accessed on July 22, 2020). Within the *Pheretima* complex, monophyly of both *Metaphire* and *Amynthas* has been controversial. Some studies have reported that these two genera are not reciprocally monophyletic (James 2005; Zhang et al. 2016). However, these analyses were conducted using a limited number of species from a limited geographic area, and therefore, require extensive analysis.

*Metaphire hilgendorfi* is one of the most common earthworms in Japan. This earthworm is widely distributed across Japan and Korea, except for the Ryukyu Islands. However, there exists a morphologically similar species *Amynthas yunoshimensis,* which is widely distributed in northern Japan. They shared a few morphological characteristics such as manicate ceca, 2 pairs of spermathecal pores between the sixth and seventh, and the seventh and eighth intersegmental furrows, and genital glands on the ventral side of the eighth and eighteenth intersegmental furrows (Hatai 1930). *A. yunoshimensis* is distinguishable from *M. hilgendorfi* owing to its smaller body size and the end of the genital papillae exceeding the setal line, yet it is difficult to identify (Hatai 1930).

In this study, we collected *M. hilgendorfi* and *A. yunoshimensis* from Tochigi, Nikko, Japan (N36.47, E139.27). Total DNA was extracted using proteinase K digestion, phenol: chloroform extraction, and isopropanol precipitation. The genomic DNA libraries were constructed using the ThruPLEX^®^ DNA-Seq Kit (Takara Bio Inc., Shiga, Japan) and sequenced to provide 301-bp paired-end reads using the MiSeq system (Illumina Inc., San Diego, CA). The raw sequence reads were cleaned up using Trimmomatic (Bolger et al. 2014) by trimming adapter sequences and low-quality ends (quality score, <30), and assembled using NOVOPlasty version 3.8.3 (Dierckxsens et al. 2017). Total data obtained were 306,926,393 bases from *M. hilgendorfi* and 122,907,420 bases from *A. yunoshimensis*, respectively. However, the sequences assembled did not include D-loop regions. Therefore, we sequenced the DNA using the Sanger method with the BigDye^®^ Terminator v3.1 Cycle Sequencing Kit (Thermo Fisher Scientific Inc., Waltham, MA) with primers M.h.-F (5′-TCAGGCCTACATTTTCTGTCTTC-3′) and M.h.-R (5′-TCCACAAAATGGGCTATTTGA-3′) for *M. hilgendorfi* and A.y.-F (5′-TTTGCTATCTGYCTAATTCAAGCCTA-3′) and A.y.-R (5′-CTCCAAAATGGGCTATTTGA-3′) for *A. yunoshimensis*. These primers were designed based on the assembly sequences. The sequences were merged with the MiSeq reads, and we obtained the complete mitochondrial genome sequences. The mitochondrial genome sequence of *M. hilgendorfi* was 15,186 bp long with an AT content of 67.21% (DDBJ/EMBL/GenBank: LC573968) and *A. yunoshimensis* was 15,109 bp long with an AT content of 64.76% (DDBJ/EMBL/GenBank: LC573969). Gene prediction and manual annotation of the mitochondrial genomes was performed using MITOS (http://mitos.bioinf.uni-leipzig.de/help.py; Bernt et al. 2013). Both genomes contained the typical gene complement of 13 protein-coding genes (PCGs), 22 tRNA genes, and 2 rRNA genes. All PCGs started with ATG and transcribed from the same direction along the DNA strand. Five PCGs (*COIII*, *ND5*, *ND4*, *ND1*, *ND2*) had incomplete stop codons, which could be completed upon post-transcriptional polyadenylation (Ojala et al. 1981).

Phylogenetic analysis was performed using the complete mitochondrial genomes of 24 Megascolecidae earthworms. The concatenated mitochondrial genomic sequences were aligned using MAFFT version 7.4.50 (Katoh and Standley 2013), and the phylogenetic tree was constructed by the maximum-likelihood method using RAxML version 8.2.10 GTRGAMMA model (1000 bootstrap replicates) (Stamatakis 2014). The phylogenetic tree revealed that *M. hilgendorfi* was the sister species of *A. yunoshimensis* (Figure 1). We found that these two species were clustered with *Amynthas jiriensis*. Our analyses rejected reciprocal monophyly between *Amynthas* and *Metaphire* according to previous studies (James 2005; Zhang et al. 2016). Therefore, the complete mitochondrial genomes of *M. hilgendorfi* and *A. yunoshimensis* reported in this study will assist in elucidating the genetic diversity, evolutionary origin, and genetic relationships for Megascolecidae earthworms.

**Figure 1.**
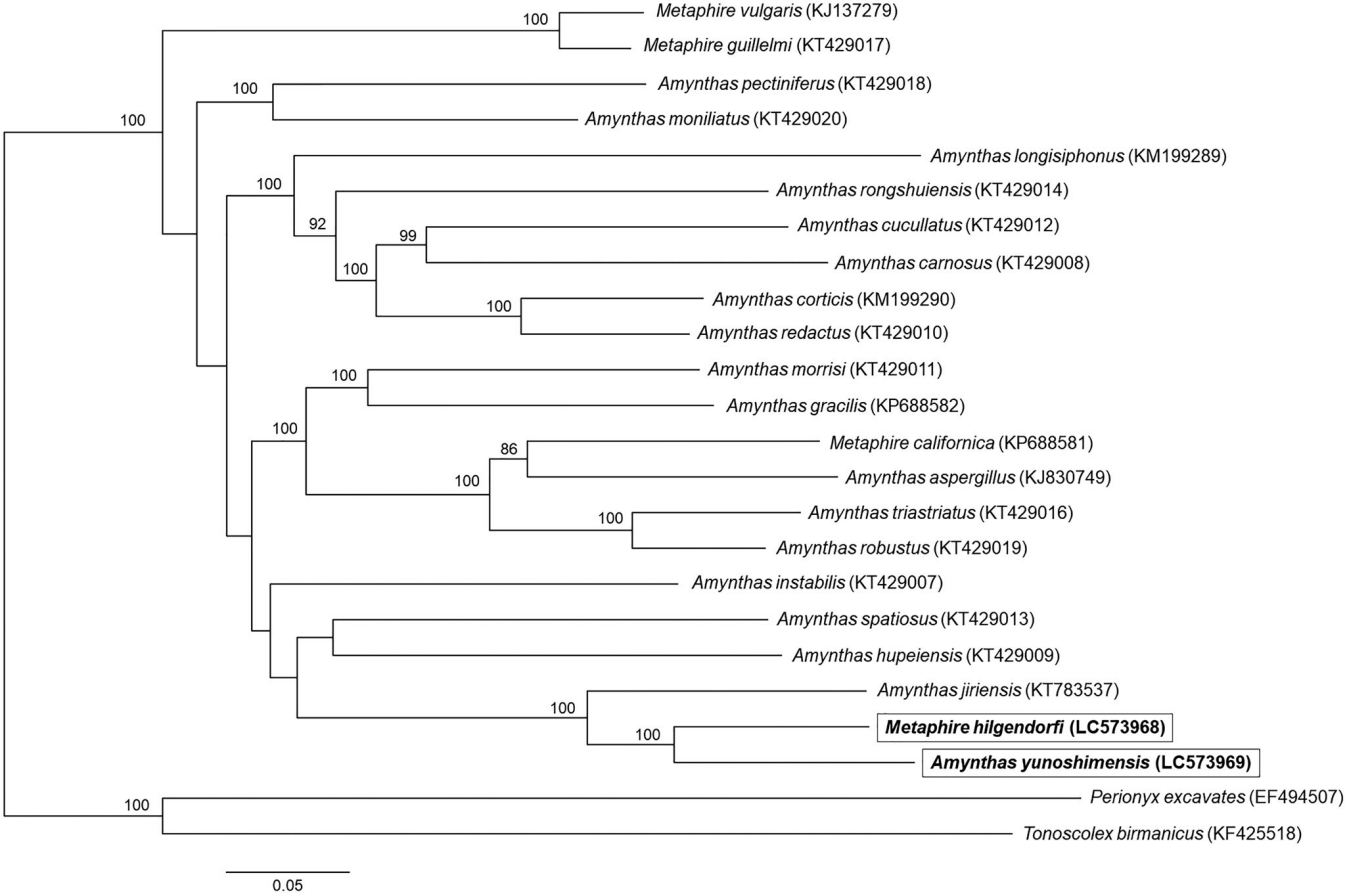
Molecular phylogenetic analysis of 24 Megascolecidae earthworms. The phylogenetic tree was constructed by the maximum-likelihood method based on the complete mitochondrial genomes. Accession numbers used in this analysis are provided next to each species name. Bootstrap values higher than 80 are shown at the nodes. Scale bar indicates the number of substitutions per site.

## Data Availability

Nucleotide sequences are available in the following URL; http://getentry.ddbj.nig.ac.jp/getentry/na/LC573968/ and http://getentry.ddbj.nig.ac.jp/getentry/na/LC573969/.
